# Supplementary material for: Eltrombopag Improves Erythroid Differentiation in a Human Induced Pluripotent Stem Cell Model of Diamond Blackfan Anemia
Source: Cells. 2021 Mar 26;10(4):734. doi: 10.3390/cells10040734 (PMC8065708; doi:10.3390/cells10040734)
Supplement: Supplementary file 1 [file cells-10-00734-s001.pdf]

cells

Article-Supplementary Materials

Eltrombopag improves erythroid differentiation in a human induced pluripotent stem cell model of Diamond Blackfan Anemia

Husam Qanash [1,2,3], Yongqin Li [1], Richard H. Smith [1], Kaari Linask [4], Sara Young-Baird [5,6], Waleed Hakami [1,2,7], Keyvan Keyvanfar [8], John S. Choy [2], Jizhong Zou [4], and Andre Larochelle [1*]

[1] Cellular and Molecular Therapeutics Branch, National Heart, Lung and Blood Institute (NHLBI), National Institutes of Health (NIH), Bethesda, MD, USA
[2] Department of Biology, Catholic University of America, Washington, DC, USA
[3] Department of Medical Laboratory Science, Applied Medical Sciences College, The University of Hail, Hail, Saudi Arabia
[4] iPSC Core Facility, NHLBI, NIH, Bethesda, MD, USA
[5] Eunice Kennedy Shriver, National Institute of Child Health and Human Development, NIH, Bethesda, MD, USA
[6] National Institute of General Medical Sciences (NIGMS), NIH, Bethesda, MD, USA
[7] Department of Medical Laboratories Technology, College of Applied Medical Sciences, Jazan University, Jazan, Saudi Arabia
[8] Clinical Flow Core Facility, NHLBI, NIH, Bethesda, MD, USA
* Correspondence: larochea@nhlbi.nih.gov; Tel: 301-451-7139

Citation: Qanash, H.; Li, Y.; Smith, R.H.; Linask, K.; Young-Baird, S.; Hakami, W.; Keyvanfar, K.; Choy, J.S.; Zou, J.; Larochelle, A. Eltrombopag improves erythroid differentiation in a human induced pluripotent stem cell model of Diamond Blackfan Anemia. *Cells* **2021**, *10*, 734. https://doi.org/10.3390/cells10040734

Received: date
Accepted: date
Published: date

Publisher's Note: MDPI stays neutral with regard to jurisdictional claims in published maps and institutional affiliations.

Copyright: © 2021 by the authors. Submitted for possible open access publication under the terms and conditions of the Creative Commons Attribution (CC BY) license (http://creativecommons.org/licenses/by/4.0/).

This document includes:

- **Supplementary Figures**

Figure S1. DBA and wild-type (WT) iPSCs display normal karyotypes

Figure S2. Absence of Sendai vector-derived antigens in DBA iPSCs

Figure S3. DBA iPSCs display characteristics of pluripotency in flow assays

Figure S4. DBA iPSCs display characteristics of pluripotency in teratoma assays

Figure S5. DBA iPSCs display ribosome biogenesis defects

Figure S6. Generation of isogenic iPSCs using CRISPR/Cas9 technology

Figure S7. Isogenic iPSCs display a normal karyotype and characteristics of pluripotency

Figure S8. Isogenic and DBA iPSCs give rise to similar percentages of total CD71+ erythroid cells

Figure S9: Reduced erythroid differentiation potential of isogenic iPSCs differentiated in the presence of EPAG

- **Supplementary Tables**

Table S1. Antibodies for flow cytometry analysis and FACS

Table S2. Primers, gRNAs and ssODN template for the generation of isogenic iPSCs

Supplementary Figures

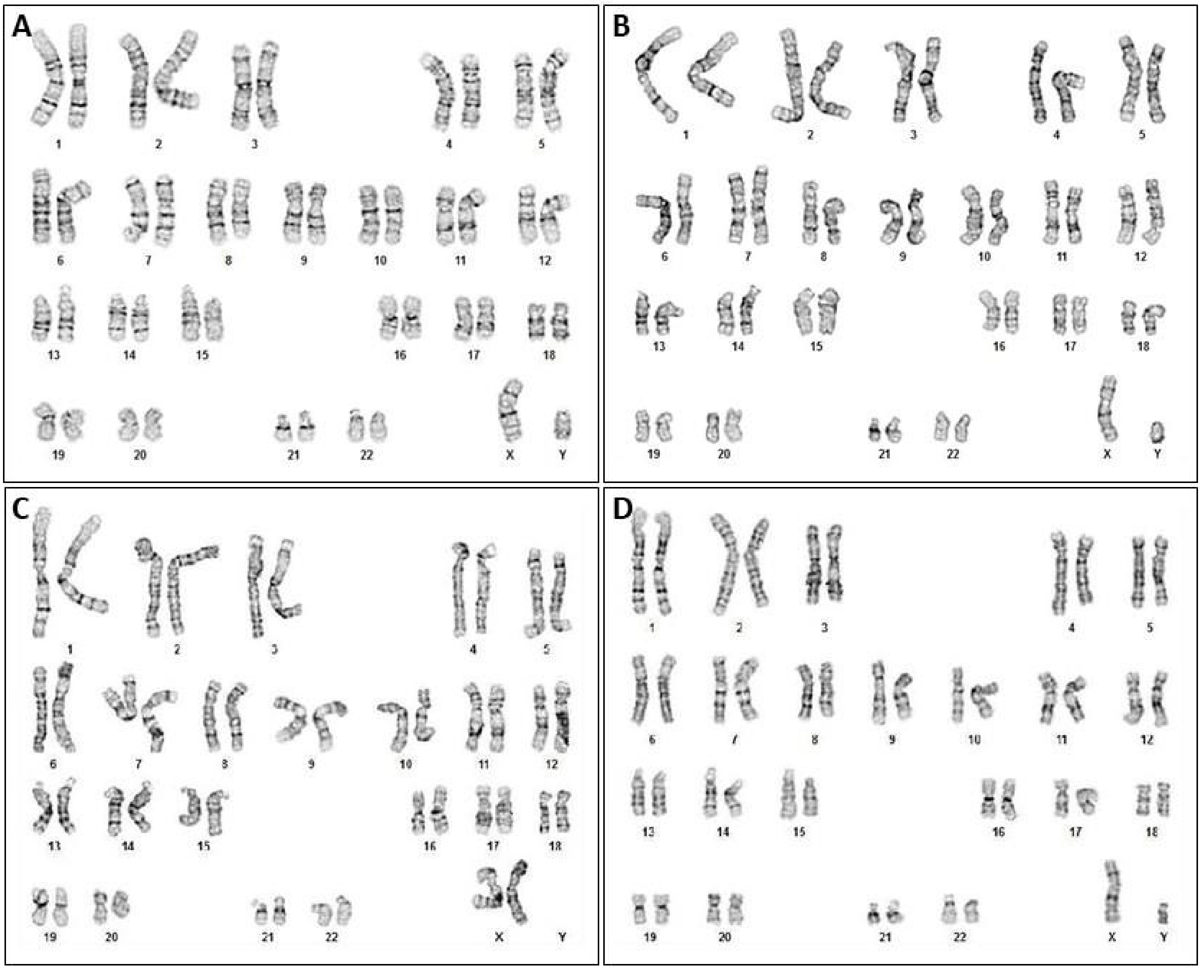

Figure S1. DBA and wild-type (WT) iPSCs display normal karyotypes. Karyotype analysis of G-banded metaphase chromosomes in WT iPSCs **(A)** and DBA iPSCs derived from patients DBA863 **(B)**, DBA869 **(C)** and DBA872 **(D)**. A normal human karyotype 46[XY] or 46[XX] was confirmed in all iPSC clones.

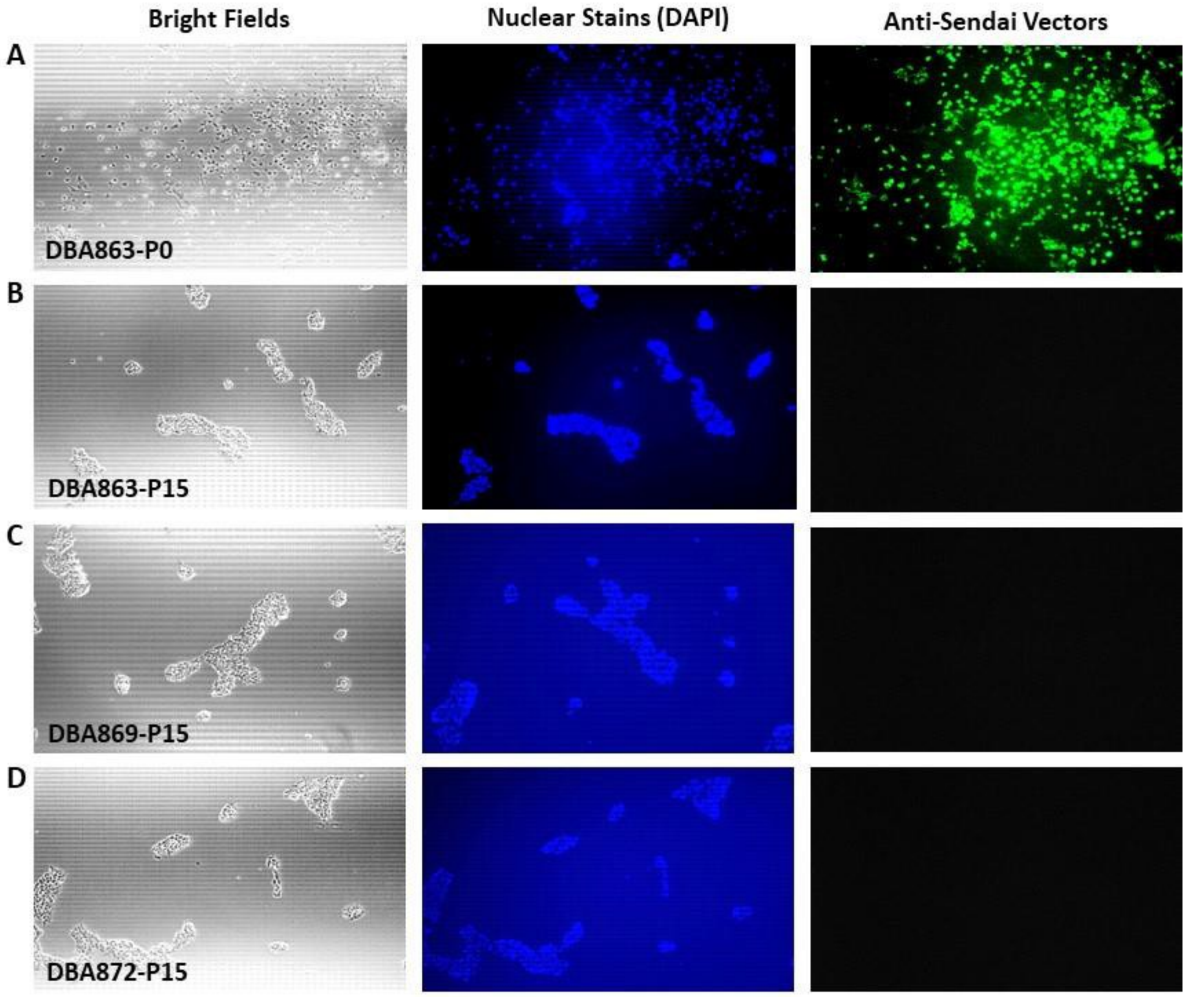

Figure S2. Absence of Sendai virus-derived antigens in DBA iPSCs. Representative immunohistochemistry stains of DBA iPSC lines. Bright fields (left panels), nuclear stains (DAPI, middle panels) and FITC-conjugated anti-Sendai vector antibody stains (right panels) are shown. (A) DBA863 iPSCS at passage 0 (P0) served as a positive control; (**B**) DBA863 iPSCs at P15; (**C**) DBA869 iPSCs at P15; (**D**) DBA872 iPSCs at P15. Sendai vectors were detected in the positive control iPSC line immediately after transduction but were undetectable after 15 passages in all DBA iPSC clones.

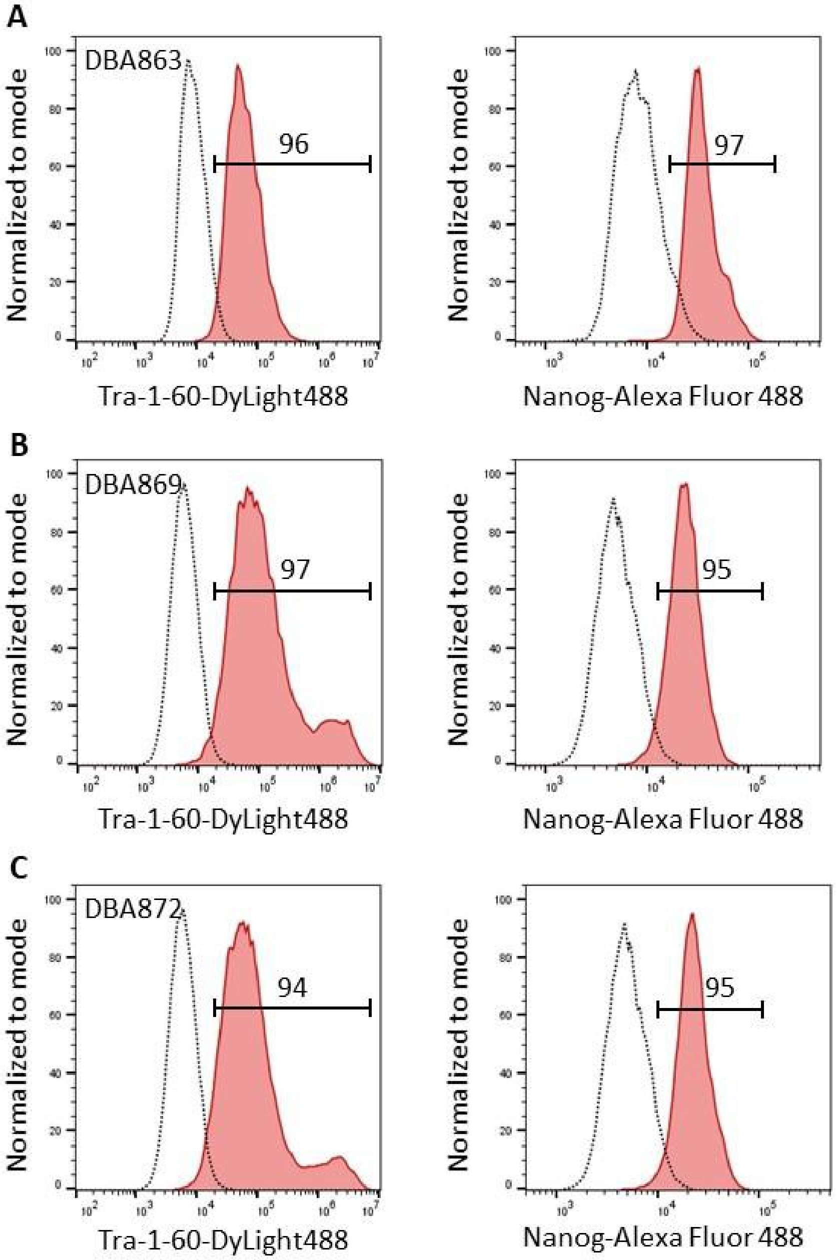

Figure S3. DBA iPSCs display characteristics of pluripotency in flow cytometric assays. Red histograms denote expression of the pluripotency markers Tra-1-60 (left panels) and Nanog (right panels) in iPSC lines derived from patients DBA863 (**A**), DBA869 (**B**) and DBA872 (**C**) relative to isotype controls (dashed lines). Numbers represent percentages of iPSCs expressing Tra-1-60 or Nanog. Peak height of each histogram was normalized to mode.

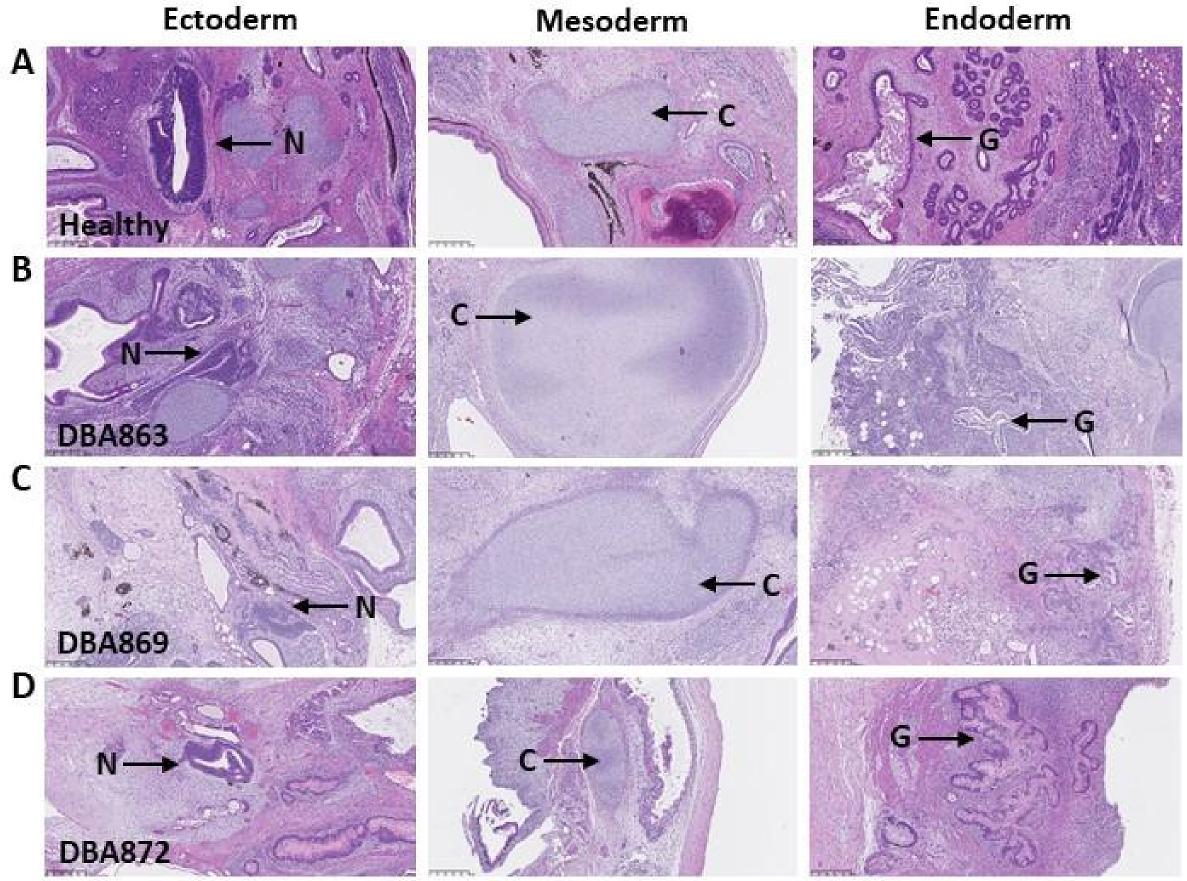

Figure S4. DBA iPSCs display characteristics of pluripotency in teratoma assays. Representative H&E stained histopathological sections of teratomas isolated from NSG mice 8 to 12 weeks after subcutaneous injection of iPSCs derived from a healthy subject (**A**) or from patients DBA863 (**B**), DBA869 (**C**) and DBA872 (**D**). Ectodermal (left panels), mesodermal (middle panels), and endodermal (right panels) tissues in teratoma sections were defined by the presence of neural rosettes (N), cartilage (C), and gut epithelium (G), respectively. Scale bars represent 250 μm.

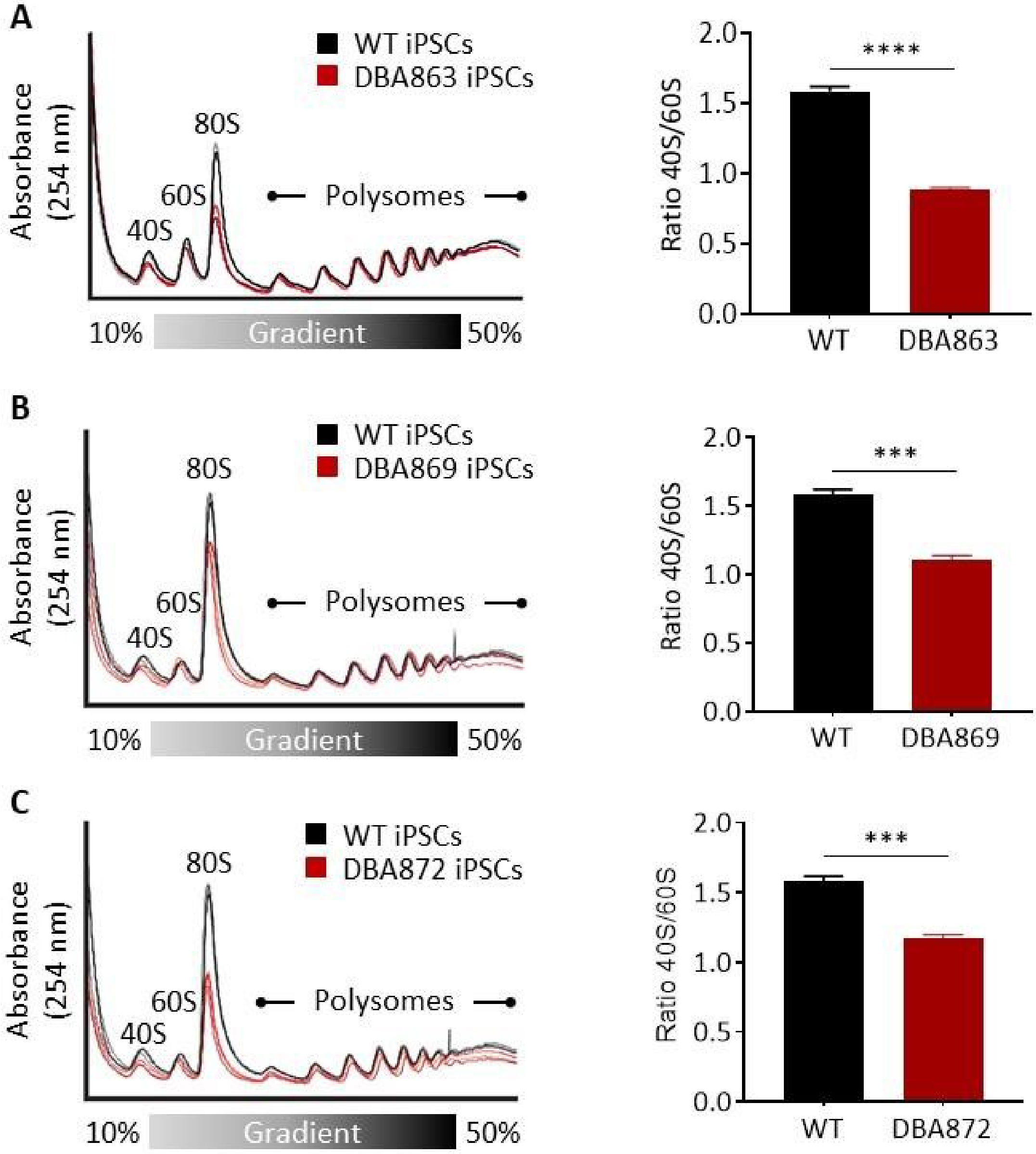

Figure S5. DBA iPSCs display ribosome biogenesis defects. Sucrose gradient (10-50%) polysome profiling analyses for wild-type (WT) control (black) and DBA863 (red) iPSCs. Peaks represent ribosomal subunits (40S and 60S), 80S monosomes, and ribosome clusters (polysomes). Polysome profiles (left panels) and 40S/60S ratios (right panels) are presented for iPSCs derived from patients DBA863 **(A)**, DBA869 **(B)** and DBA872 **(C)**. In right panels, data are presented as mean ± SEM. Unpaired t-test, *** $p \leq 0.001$, **** $p \leq 0.0001$ (n=3).

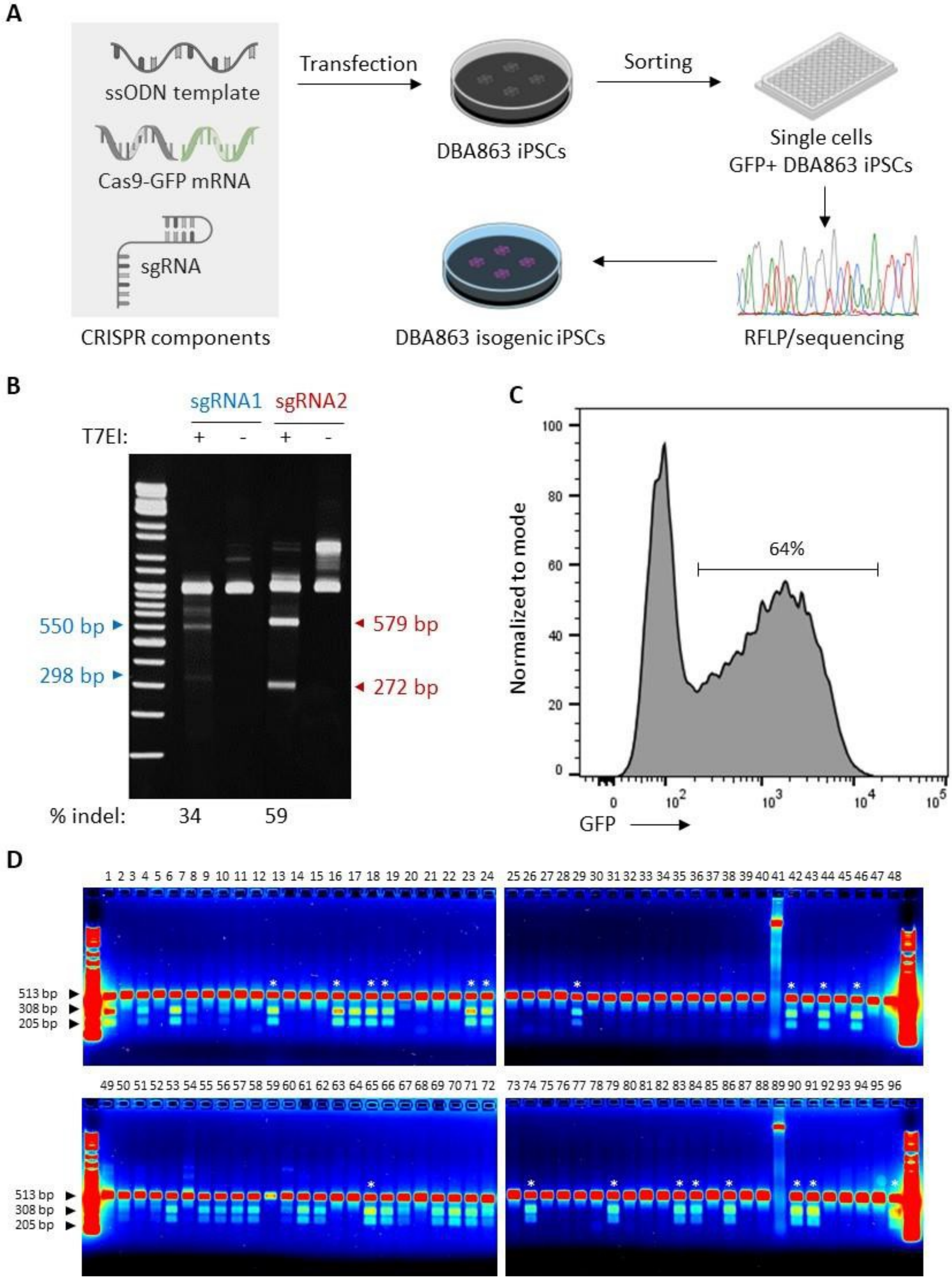
A
ssODN template
Cas9-GFP mRNA
sgRNA
CRISPR components
Transfection
DBA863 iPSCs
Sorting
Single cells
GFP+ DBA863 iPSCs
RFLP/sequencing
DBA863 isogenic iPSCs
B
sgRNA1
sgRNA2
T7EI:
+
-
+
-
550 bp
298 bp
579 bp
272 bp
% indel:
34
59
C
Normalized to mode
64%
GFP
D
513 bp
308 bp
205 bp

Figure S6. Generation of isogenic iPSCs using CRISPR/Cas9 technology. **(A)** Workflow for the generation of isogenic iPSCs. The process includes 3 steps: 1) Lipid-mediated transfection of DBA iPSCs with CRISPR/Cas9 components (mRNA encoding Cas9-GFP fusion protein, sgRNA, and ssODN template); 2) Sorting and re-plating of GFP+ iPSCs into 96-well plates; 3) Identification of corrected isogenic iPSCs by restriction fragment length polymorphism (RFLP) and sequencing. **(B)** Identification of an optimal sgRNA sequence. Two candidate sgRNAs (DBA sgRNA1 and DBA sgRNA2) were selected and their respective efficiencies tested using a T7 endonuclease I (T7EI) cleavage assay. Each cleavage reaction was conducted in the presence (+) or absence (-) of T7EI and run on a 4-20% polyacrylamide TBE gel. Predicted cleavage product lengths are indicated adjacent to the corresponding bands on the gel. The percentages of indel formation (% indel) were calculated using ImageJ software. DBA sgRNA2 displayed significantly higher indel formation compared to DBA sgRNA1; this guide was selected for subsequent gene correction experiments. **(C)** Representative flow cytometry histogram of GFP+ iPSCs obtained 24 hours after transfection of Cas9-GFP mRNA and other CRISPR/Cas9 components. Peak height of histogram was normalized to mode. **(D)** AscI restriction enzyme digestion of a 513 bp PCR amplicon of the targeted RPS19 locus. Each lane of the agarose gel represents a cellular isolate with a total of 60 isolates confirmed as single cell iPSC clones. The highlighted lanes (*) represent gene corrected single cell isogenic iPSC clones corresponding to an overall correction efficiency of 19/60 (32%). Lanes 41 and 89 represent control AscI digestion of genomic DNA isolated from uncorrected DBA863 iPSCs.

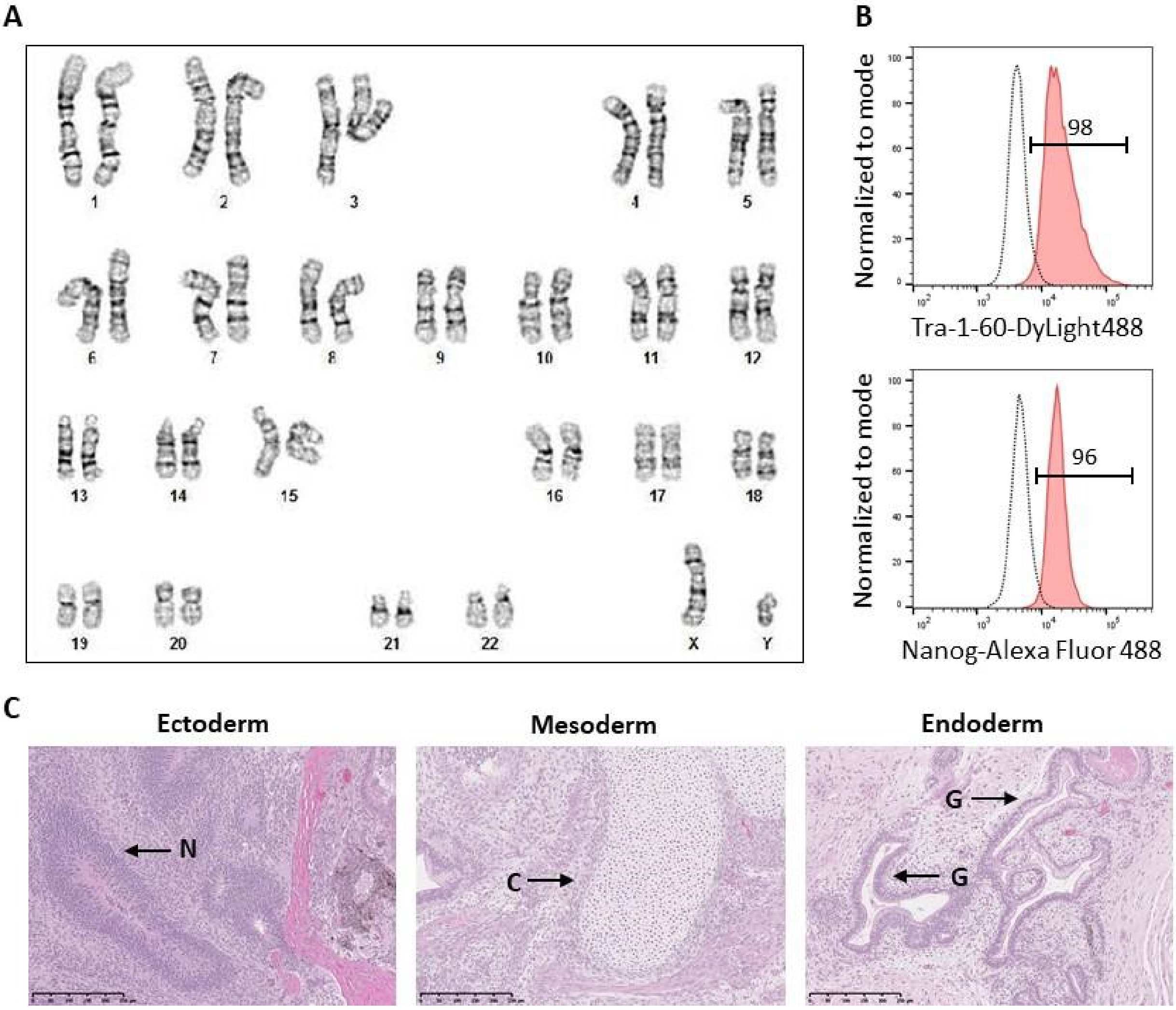

Figure S7. Isogenic iPSCs display a normal karyotype and characteristics of pluripotency. **(A)** Karyotype analysis of G-banded metaphase chromosomes in isogenic iPSCs; a normal human karyotype 46[XY] was confirmed. **(B)** Flow cytometric evaluation of expression of the pluripotency markers Tra-1-60 (top panel) and Nanog (bottom panel) in isogenic iPSCs (red histograms) relative to isotype controls (dashed line). Numbers represent percentages of iPSCs expressing Tra-1-60 or Nanog. Peak height of each histogram was normalized to mode. **(C)** Representative H&E stained histopathological sections of teratomas isolated from NSG mice 8 to 12 weeks after subcutaneous injection of isogenic iPSCs. Ectodermal (left panel), mesodermal (middle panel), and endodermal (right panel) tissues in teratoma sections were defined by the presence of neural rosettes (N), cartilage (C), and gut epithelium (G), respectively. Scale bars represent 250 µm.

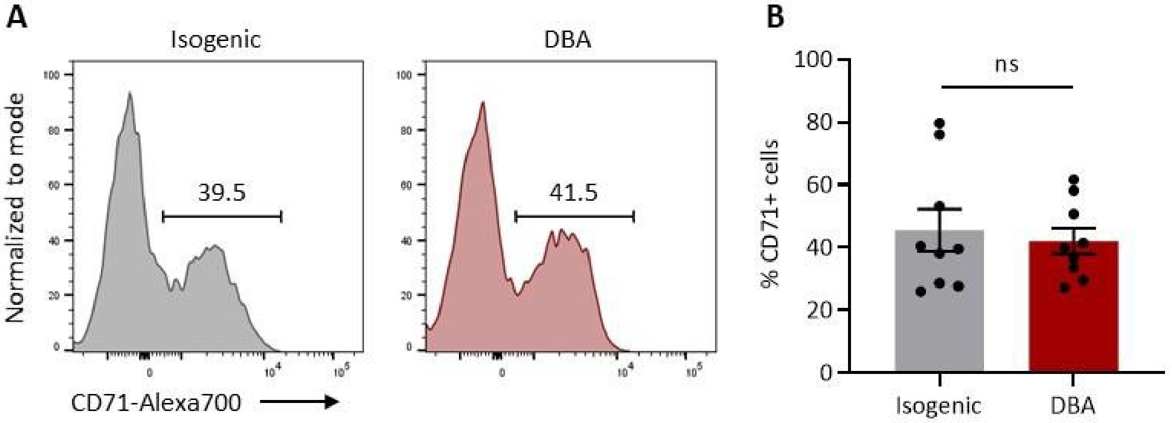

Figure S8. Isogenic and DBA iPSCs give rise to similar percentages of total CD71+ erythroid cells. **(A)** Representative flow cytometry histograms depicting percentages of CD71+ erythroid cells within the live-gated hematopoietic CD43+CD45+/- population at culture day 21 of isogenic and DBA iPSC differentiation. Peak height of each histogram was normalized to mode. **(B)** Percentages of CD71+ cells within the live-gated hematopoietic CD43+CD45+/- population at culture day 19 or 21 of isogenic and DBA iPSC differentiation (n = 9). In panel B, data are presented as mean ± SEM. Unpaired t-test, ns: not significant.

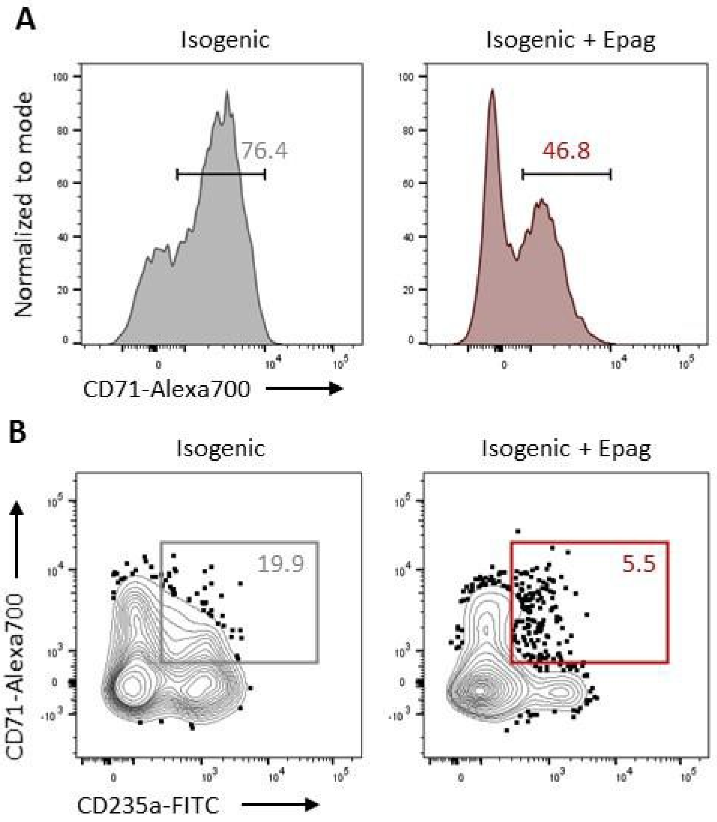

Figure S9. Reduced erythroid differentiation potential of isogenic iPSCs differentiated in the presence of EPAG. **(A)** Representative flow cytometry histograms depicting percentages of CD71+ erythroid cells within the live-gated hematopoietic CD43+CD45+/- population at culture day 21 of isogenic iPSC differentiation in the presence or absence of EPAG. Peak height of each histogram was normalized to mode. In two independent experiments, we observed a fold reduction of 1.6 ± 0.1 in total CD71+ erythroid cells at day 19-21 of differentiation. **(B)** Representative flow cytometry contour plots depicting percentages of mature CD45- erythroblasts co-expressing CD71 and CD235a at culture day 21 of isogenic iPSC differentiation in the presence or absence of EPAG. In two independent experiments, we observed a fold reduction of 2.9 ± 0.9 in CD71+CD235a+ late-stage CD45- erythroblasts at day 19-21 of differentiation.

Supplementary Tables

Table S1 | Antibodies for flow cytometry analysis and FACS

Antigen	Fluorochrome	Company/Catalogue #	Species	/10^6 cells
CD235a	FITC	BioLegend/349103	Mouse anti-human	2 µL
CD34	PE-Cy7	BD Pharmingen/560710	Mouse anti-human	10 µL
CD43	BV711	BD Pharmingen/743614	Mouse anti-human	5 µL
CD45	V450	BD Pharmingen/560367	Mouse anti-human	5 µL
CD71	AF700	BD Pharmingen/563769	Mouse anti-human	10 µL
EPOR	PE	R&D systems/FAB307P	Mouse anti-human	15 µL
Nanog	AF488	Millipore FCABS352A4	Rabbit anti-human	1:50
Tra-1-60	DyLight488	Thermo/MA1-023	Mouse anti-human	0.4 µg
Viability	7-AAD	Thermo/00-6993-50	N/A	5 µL

Table S2 | Primers, gRNAs and ssODN template for the generation of isogenic iPSCs

Name	Gene	Company	Sequence
DBA sgRNA1	RPS19	Synthego	5′-UGUACCUCCGGGUGGCGCU-3′
DBA sgRNA2	RPS19	Synthego	5′-GAGGUACAGGUGCUGCGCUG-3′
ssODN	RPS19	IDT	GAATTAGCTGTTTACACACAAGGAATTGTTTACCTGAGACCTT GATCAAGACCCTAATCTCCCTCTCACACTACCCCAGCTT**CGAC** GGCGCGCCACCTGTACCTCCGGGGTGCGCTGGGGTTGGCTCC*
DBA F primer	RPS19	IDT	5′-AGCAGTGCTGCTGGGATAGT-3′
DBA R primer	RPS19	IDT	5′-GCTTCTCAAAAAGCCACCTG-3′
Isogenic F primer	RPS19	IDT	5′-GGCTCCACCCCTACATAACC-3′
Isogenic R primer	RPS19	IDT	5′-CATCAATGCAGCCCCCTCTA-3′

*Red: Mutation correction base; Blue: silent mutation to abolish the PAM sequence; Green: silent mutation to introduce an AscI restriction enzyme digestion site (underlined). **Bold**: altered PAM sequence after correction.
